# Clearance of apoptotic cells by neutrophils in inflammation and cancer

**DOI:** 10.1038/s41420-024-01809-7

**Published:** 2024-01-13

**Authors:** Cristiano Ramos, Rudolf Oehler

**Affiliations:** Department of General Surgery, Division of Visceral Surgery, Medical University of Vienna, Vienna, Austria

**Keywords:** Translational research, Experimental models of disease

## Abstract

When a cell dies of apoptosis, it is eliminated either by neighbouring cells or by attracted professional phagocytes. Although it was generally believed that neutrophils also have the ability to perform efferocytosis, their contribution to the clearance of apoptotic cells was considered less important compared with macrophages. Therefore, this ability of neutrophils remained unexplored for a long time. Over the past decade, it has been shown that during inflammation, neutrophils contribute significantly to the clearance of apoptotic neutrophils that accumulate in large numbers at the site of tissue damage. This “neutrophil cannibalism” is accompanied by inhibition of pro-inflammatory activities of these cells, such as respiratory burst and formation of neutrophil extracellular traps (NETs). Furthermore, efferocytosing neutrophils secrete anti-inflammatory mediators and mitogens including hepatocyte growth factor (HGF), fibroblast growth factor 2 (FGF2), vascular endothelial growth factors (VEGF), and transforming growth factor beta (TGFβ). Thus, efferocytosis by neutrophils is involved in resolution of inflammation. Recent research indicates that it plays also a role in cancer. Many different solid tumours contain aggregates of dead tumour cells that have undergone spontaneous apoptosis. Their extent correlates with poor clinical outcome in most cancer types. These clusters of apoptotic tumour cells are strongly infiltrated by tumour-associated neutrophils (TANs) that acquired an anti-inflammatory and pro-resolving polarization state. This review summarizes the potential consequences discussed in the current literature. Although the picture of the role of efferocytosis by neutrophils in inflammation and cancer is becoming clearer, many questions are still unexplored.

## FACTS

- Apoptotic cells release “find-me” signals which attract predominantly neutrophils.
- Neutrophils accumulate in areas of tissue damage and apoptotic tumour cells.
- After engulfment of apoptotic cells, neutrophils block respiratory burst and NETosis.
- Efferocytosing neutrophils secrete a variety of soluble mediators such as cytokines, chemokines, and mitogens which create a pro-resolving and tumorigenic microenvironment.

## OPEN QUESTIONS

- Several of the molecules known to be involved in the process of efferocytosis in macrophages are also expressed in neutrophils, but for many of them there is still a lack of evidence that they also fulfil this function there.
- The exact mechanism by which neutrophils adopt a regenerative and tumourigenic phenotype after the uptake of apoptotic cells is still largely unexplored.

## Neutrophils in inflammation

Neutrophils represent the first line of cellular innate immune response to infection and tissue damage. Recent evidence indicate that this short-lived myeloid cell population exhibits a great phenotypic and functional diversity [1]. It not only plays an important role in triggering inflammation in reaction to pathogens, but also contributes to its subsequent resolution after their clearance. Neutrophils accumulate quickly at the site of tissue damage through a multi-step process called “neutrophil swarming” [2]. Damage-associated molecular patterns (DAMPs) activate resident cells to release short-range chemoattractants for neutrophils. Pioneer neutrophils from around the damage site migrate to the tissue injury within minutes. The contact with pathogen-associated molecular patterns (PAMPs) stimulates them to deploy a plethora of antimicrobial weapons [3]. They form neutrophil extracellular traps (NETs) to entrap invading pathogens. They release a variety of antimicrobial and pro-inflammatory molecules from their granules and produce reactive oxygen species to kill bacteria. Finally, they clear pathogens by phagocytosis. Neutrophil-derived leukotriene B4 (LTB4) enhances the radius of recruitment of further neutrophils from distant tissue sites [2]. Notably, neutrophils also support the resolution of inflammation right from the start. They release anti-inflammatory, resolving and angiogenic mediators such as IL-10, transforming growth factor β (TGFβ), lipoxin 4A, resolvins, protectins, defensins, and vascular endothelial growth factor (VEGF) [4]. Neutrophils that have engulfed pathogens die through phagocytosis-induced apoptosis [5]. Cytokine receptors such as IL-1R on their surface, which are no longer functional, scavenge their pro-inflammatory ligands from the microenvironment [6]. Neighbourhood macrophages that phagocytose dying neutrophils adopt an anti-inflammatory, resolving and reparative M2-like phenotype [7–9]. Such resolving mechanisms begin to gain the upper hand as soon as the infection is pushed back. The accumulation of neutrophils at the site of tissue damage thus enables the restructuring of the extracellular matrix, the formation of dense aggregates that seal the wound tightly, and finally the initiation of tissue repair processes. It must be emphasized that signals of tissue damage are sufficient to trigger the attraction of neutrophils, which accordingly can also be observed in response to sterile injury without pathogens [3].

## Efferocytosis by neutrophils

Especially during the early phase of neutrophil swarming, the number of resident macrophages is still very low and probably insufficient to clear all apoptotic neutrophils. Kristina Rydell-Törmänen showed in a mouse model of sterile lung inflammation that almost 50% of neutrophils at the side of injury have phagosomes that contained material from other neutrophils [10]. The authors termed this process “neutrophil cannibalism”. The efferocytotic capacity of neutrophils is similar to that of blood derived DCs, but clearly lower as compared to blood-derived macrophages [11]. It increases in response to pro-inflammatory cytokines such as TNF-α, interferon-gamma (IFN-γ) and granulocyte-macrophage colony-stimulating factor (GM-CSF) and to ligands of TLR2 (Malp2, Pam3CSK4), TLR4 (LPS), TLR7/TLR8 (R848), and TLR9 (ODN 2006) [11, 12]. The efferocytotic ability of neutrophils is not exclusively limited to apoptotic conspecifics but also includes remnants of other cell types.

### Detection of apoptotic cells

**Fig. 1:**
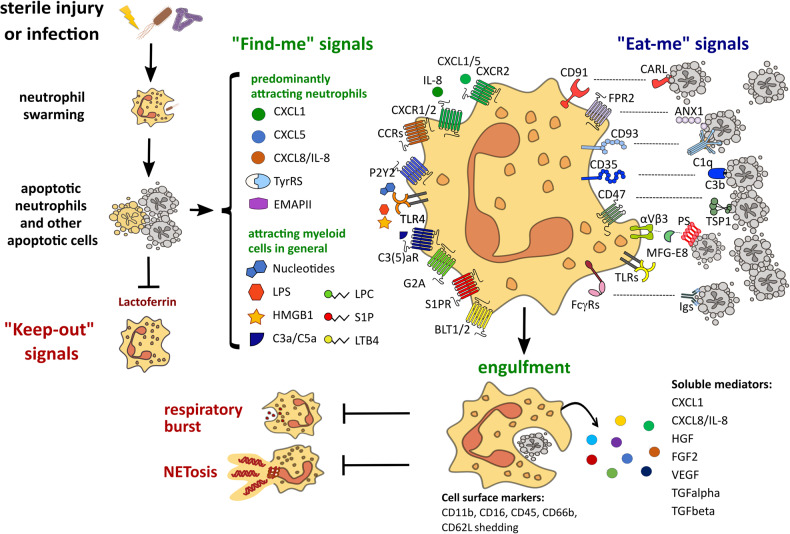
Efferocytosis by neutrophils. In response to sterile injury or local infection, neutrophils migrate to the site of tissue damage in a multistep process termed “neutrophils swarming”. Neutrophils undergo apoptosis after phagocytosis of invading pathogens and contribute to local apoptotic cells. Apoptotic cells release “keep-out” signals as well as “find-me” signals, many of which are strong chemoattractants for neutrophils. Neutrophils detect “find-me” signals on the surface of apoptotic cells and engulf the cell remains. This leads to a blockade of signalling pathways responsible for respiratory burst and NETosis. Furthermore, efferocytosing neutrophils expose specific cell surface activation markers and secrete a variety of soluble mediators.

Apoptotic cells in general release various “find-me” signals that specifically attract neutrophils, including CCL3, CXCL1, CXCL5, CXCL8/IL8, tyrosyl tRNA synthetase (TyrRS) and endothelial monocyte activating polypeptide II (EMAPII) [13–15] (Fig. 1). This suggests that neutrophils may be intentionally recruited to help clear apoptotic cell debris. It has to be noted that apoptotic cells release also lactoferrin, which inhibits neutrophil migration [16]. However, this “keep-out” signal seems not to be sufficient to antagonize other chemoattractants in vivo. Garg and co-workers induced apoptotic cell death in a lung carcinoma cell line before injecting them intradermally into the mice ear pinna [17]. Cells exposed to the immunogenic apoptosis inducer mitoxantrone stimulated rapid recruitment of neutrophils, which in comparison to other leucocyte subsets constituted the predominant immune cell population accumulating at sites of apoptosis. Similarly, neutrophils accumulate at sites of apoptotic hepatocytes of patients with hepatocellular carcinoma [18].

The predominant “eat me” signal on the surface of apoptotic cells is phosphatidylserine (PS). There is an extensive literature on the different mechanisms that macrophages use to detect PS-positive cell debris (reviewed in [8, 19]). They include directly binding receptors such as adhesion G protein-coupled receptor B1 (ADGRB1), stabilin-2 or T-cell membrane protein 4 (TIM-4). Furthermore, PS is also detected indirectly via soluble “bridging factors” like growth-arrest-specific gene-6 (GAS6) or Milk fat globule-EGF factor 8 protein (MFG-E8), which bind to PS and are then themselves detected by specific receptors on the macrophage. The MFG-E8 receptor αVβ3 integrin is also highly expressed in neutrophils [20]. However, neutrophils do not express any direct PS receptor (ADGRB1, stabilin-2, or TIM-4) or any receptor of GAS6. Besides PS, there are also other ‘eat-me’ signals exposed on apoptotic cells, including calreticulin, annexin A1, thrombospondin 1 binding sites, and complement proteins C1q or C3b binding sites [21]. They are recognized by CD91, formyl peptide receptor 2, CD47, CD93, and CD35, respectively. All of them are highly expressed in neutrophils [22–26]. However, their functional role in efferocytosis by these cells is still unexplored.

### Clearance of apoptotic cells

The subsequent events in efferocytosis comprise the engulfment of apoptotic cellular corpses, followed by the formation and maturation of the phagosome, culminating with the degradation of the cargo within the phagolysosomal compartment. The molecular mechanisms of this tightly regulated multi-step process have been investigated in detail in macrophages (reviewed in [27]). After activation of “eat-me” receptors, the submembranous actin cortex undergoes specific rearrangements which activates the Rho family of small GTPase RAC1 and promotes the formation of a phagosome [27, 28]. The processing of engulfed cellular cargo requires a non-canonical LC3-asscociated phagocytosis (LAP) [29]. LAP represents a specialized mechanism that utilizes components of the autophagic machinery to enhance the degradation of phagocytosed material in an immunologically silent manner [30]. LAP is triggered by the recruitment of LC3 (microtubule-associated protein 1A/1B-light chain 3) proteins to the single-membrane phagosomes (or LAPosome). For that, PI3KC3 complex needs to be assembled, which consists of Rubicon, vps34, beclin-1, and vps15. This complex converts the LAPosome-bound phosphatidylinositol into the signalling lipid phosphatidylinositol 3-phosphate (PI3P) [27, 28]. The PI3P-coated LAPosome stabilizes the NOX2 complex which is responsible for ROS generation, leading to LC3 ligation machinery activation and LC3-II recruitment to the LAPosome [31]. This last step facilitates the LAPosme-lysosome fusion, resulting in a rapid degradation of the cargo. Recently, Prajsnar et al. identified the LAP machinery in neutrophils, but unfortunately its activation upon efferocytosis of apoptotic cells has not been investigated [32].

Cunha et al. noted that engulfment of apoptotic corpses per se does not result in immunosuppression, but it is rather the subsequent accumulation of digested products which induces immune tolerance [33]. The phagocytes overload with lipids and cholesterol stimulates the activation of nuclear steroid receptors from the liver X receptors (LXRs) and peroxisome proliferator-activated receptors (PPARs) families [34, 35]. Apart from mediating lipid homoeostasis, LXRs and PPARs induce the clearance of apoptotic cells via expression of phagocytic receptors and opsonins, resembling a positive feedback loop. The anti-inflammatory effects attributed to efferocytosis are also mediated by these pathways, by promoting upregulation of the anti-inflammatory cytokines TGFβ and IL-10 whereas the pro-inflammatory cytokines TNFα, IL-1β, and IL-6 are downregulated [19]. A similar response has been observed in neutrophils that had phagocytosed apoptotic neutrophils [11, 36]. They showed an elevated expression of anti-inflammatory TGFβ and of neutrophil chemoattractants CXCL1 and CXCL8/IL8, and a lower secretion of pro-inflammatory cytokines TNFα and CXCL10/IP-10. Furthermore, they downregulate respiratory burst due to a reduced phosphorylation of p38 MAPK and PKCδ, the kinases involved in NADPH oxidase activation [37]. Incubation with anti-TGFβ1 antibodies restores respiratory burst [36]. The inhibitory effect of neutrophil cannibalism on respiratory burst is exploited by invading bacteria to their own advantage. For instance, *Leishmania major*-infected neutrophils acquire enhanced capacity to engulf apoptotic cells. The uptake of apoptotic cells inhibits respiratory burst, protecting thereby the bacteria [37]. Manfredi et al. found that MPO and elastase are translocated into phagolysosomes during the process of efferocytosis to facilitate cargo degradation, making these enzymes unavailable for participating in chromatin decondensation – a prerequisite for NET formation [38]. Thus, neutrophilic efferocytosis impedes NETosis and primes these cells towards a non-inflammatory and resolving phenotype. We could demonstrate recently that neutrophils engulf apoptotic cell-derived extracellular vesicles (aEV) from hepatocytes and several cancer cell lines [39]. This is associated with an increase of cell surface activation markers CD11b, CD16, CD45, CD66b, CD62L, and secretion of various mitogens, including hepatocyte growth factor (HGF), fibroblast growth factor 2 (FGF2), VEGF, and transforming growth factor alpha (TGFα). Neutrophils express HGF mRNA and store the active protein in secretory vesicles and gelatinase granules [40]. The release of HGF and other mitogens in response to aEV results in an elevated metabolic activity and proliferation of co-cultured hepatocytes [39]. This indicates that efferocytosing neutrophils induce tissue regeneration in response to an uptake of apoptotic cells. Strong corroboration for this hypothesis comes from an observation in patients undergoing partial hepatectomy, a surgical procedure that results in massive local apoptosis at the resection margins of the remaining liver lobes [39]. Free HGF as well as neutrophil-bound HGF in the circulation of these patients correlate with the degree of apoptosis. Notably, higher levels of HGF are associated with improved liver regeneration.

## Neutrophils in cancer

**Fig. 2:**
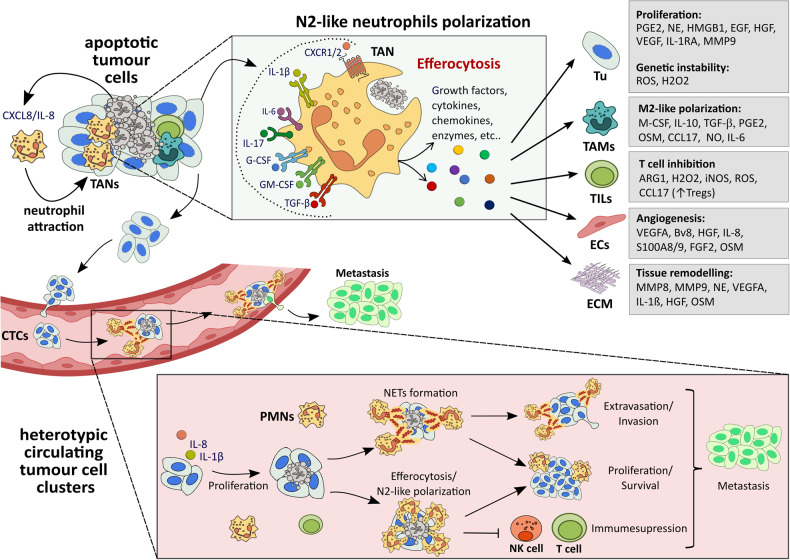
Efferocytosis by neutrophils in cancer. Spontaneous or therapy-induced tumour apoptosis leads to attraction of tumour-associated neutrophils (TANs). They are exposed to tumour-derived factors (dotted line) and engulf apoptotic cell remains. Both polarizes efferocytosing neutrophils towards an anti-inflammatory and pro-resolving N2-like phenotype. These TANs secrete numerous soluble mediators, which modulate tumour cells (Tu), tumour-associated macrophages (TAMs), tumour infiltrating lymphocytes (TILs), endothelial cells (ECs) and the extracellular matrix (ECM) in a pro-tumourigenic way. In addition, cancer may intravasate into adjacent vessels resulting in circulating tumour cells (CTCs). The blood stream is a harsh environment and many of CTCs have a short half-life. CTCs may form clusters with high abundant blood cells such as platelets or neutrophils (polymorphonuclear leucocytes or PMNs). They form NETs protecting CTCs and supporting extravasation and metastasis. Apoptotic CTCs within clusters are expected to bind neutrophils for efferocytosis, which would further support CTC survival and proliferation. However, experimental proof for this role of efferocytosis by neutrophils is still pending.

Fridlender et al. identified two distinct populations of tumour-associated neutrophils (TANs): anti-tumourigenic N1 and pro-tumourigenic N2 TANs [41]. The latter type prevails in many human cancers [42]. It releases a variety of cytokines, chemokines, and growth factors that promote tumour cell survival and proliferation, such as prostaglandin E2 (PGE2), CCL17, interleukin-6 (IL-6), tumour necrosis factor-alpha (TNF-α), VEGF, and epidermal growth factor (EGF) (Fig. 2) [43]. N2 TANs also secrete collagenase (MMP8) and gelatinase B (MMP9), which facilitate the invasion of tumour cells by remodelling the extracellular matrix [44]. In addition, their arginase-1 (ARG1) degrades extracellular arginine, which dampens the proliferation of T cells [45]. Thus, N2 TANs resemble in many respects to neutrophils after uptake of apoptotic cells.

Spontaneous apoptosis of single tumour cells can be observed in many treatment-naive patients. It was shown already more than 25 years ago in prostate cancer that an elevated frequency of tumour cell apoptosis correlates with a higher 5-years progression rate [46]. Similarly, colorectal cancer patients with a higher number of apoptotic cancer cells have a worse overall survival [47]. Table 1 summarizes numerous studies investigating the relationship between cancer apoptosis rate and clinical outcome in 18 different cancer types. A positive association between apoptosis and poor prognosis was found in most cancers types. Only thyroid carcinoma, neuroblastoma, and glioblastoma showed an inverse relationship. Thus, tumour cell apoptosis promotes the progression of remaining viable tumour cells. Apoptotic cells release the growth factor FGF-β, PGE2, and VEGF, which have a direct promoting effect on the proliferation of adjacent tumour cells (recently reviewed in [48]). However, there is strong evidence that the pro-tumourigenic effect of apoptosis is mainly mediated by the phagocyte response during apoptotic cell clearance [49]. Most studies focussed on macrophages, whereas the contribution of efferocytosing neutrophils to tumour growth is much less investigated. Dead tumour cells are not equally distributed throughout the tumour tissue. Many solid cancers show dense cribriform nests or pseudoluminal structures with central aggregates of disintegrated dead tumour cells [50]. We found recently in colorectal cancer patients that such massive dead cell accumulations stain positive for caspase-cleaved cytokeratin 18 and CXCL8/IL-8, indicating that they derive from apoptotic tumour cells, which release a neutrophil chemoattractant (Fig. 2) [15]. Accordingly, the great majority of aggregates is highly infiltrated with neutrophils and anti-inflammatory polarized TAMs. Blocking the apoptotic cell-derived CXCL8/IL-8 prevents neutrophil-induced anti-inflammatory macrophage polarization. These data fit to the above-proposed concept that neutrophils play a major role in efferocytosis in cases of massive accumulations of apoptotic dead cell remnants.

**Table 1:** Correlative assessment of spontaneous apoptosis in situ with cancer patients’ outcome in several tumour types.

Tumour type	Method to detect apoptosis	OS^a^	Comment	Refs.
Non-Hodgkin’s lymphoma	TUNEL	↓	High grade vs low grade	[59]
	TUNEL + H&E	↓	High versus low tumour cell turnover	[60]
Breast carcinoma	IHC/Caspase-3	↓	OE is 75% of invasive BC	[61]
	H&E	↓*	*Recurrence-free survival	[62]
	TUNEL + H&E	↓	>0.50% (shorter OS)	[63]
	FC + H&E	↓		[62]
	IHC/Cleaved caspase-3	↓		[64]
Ovarian carcinoma	IHC/HtrA20	↑	Increased response to chemotherapy	[65]
	ELISA (Smac/DIABLO)	↑	Serum concentrations	[66]
	FC + IHC (Caspase-3)	↑	Metastasis	[67]
	IHC /Cleaved caspase-3	↓		[68]
	TUNEL	↓	ovarian serous carcinoma	[69]
Cervical cancer	IHC/Cleaved caspase-3	↓		[68]
Colorectal carcinoma	IHC/Cleaved caspase-3	↓		[68]
	IHC (M30)	↓	Higher turnover tumours	[47]
	TUNEL + H&E	↓		[70]
	TUNEL + H&E	↑		[71]
Lung carcinoma	TUNEL + H&E	↓	Non-small cell lung carcinoma	[72]
	TUNEL	↓	Non-small cell lung carcinoma	[73]
Gastric carcinoma	IHC /Cleaved caspase-3	↓		[68]
	TUNEL	↑	advanced gastric carcinoma	[74]
Prostate carcinoma	H&E	↓		[75]
	H&E	↓*	*Actuarial progression rate at 5 years	[46]
	TUNEL	↓*	*Disease recurrence	[76]
Thyroid carcinoma	TUNEL	↑	papillary thyroid carcinoma (PTC)	[77]
Bladder carcinoma	H&E	↓		[78]
	IHC	↓	Invasive transitional cell carcinoma	[79]
Pancreatic carcinoma	TUNEL	↓	Higher AI in undifferentiated vs. differentiated cancers	[80]
	TUNEL	↓		[81]
Salivary glands	TUNEL	↓		[77]
Hepatocellular carcinoma	H&E	↑	low growth index	[82]
	H&E	↓*	*Disease-free survival	[83]
Neuroblastoma	TUNEL	↑		[84]
Mesothelioma	TUNEL + H&E	↓		[85]
	TUNEL	↓	Pleural mesothelioma	[86]
Tongue carcinoma	TUNEL	↓	Early stage squamous carcinoma	[87]
Laryngeal carcinoma	TUNEL	↓	Squamous cell carcinoma	[88]
Glioblastoma	TUNEL + H&E	↑		[89]

Patient-derived histological samples were analyzed by using either DNA end-labelling techniques (TUNEL), plain morphology combined with hematoxylin and eosin (H&E), immunohistochemistry (IHC), flow cytometry (FC), or enzyme-linked immunosorbent assay (ELISA). Clinical studies in which patients were treated before assessment were excluded from the analysis.

^a^Overall survival (if not otherwise indicated in the comments field) at increased apoptosis.

Interestingly, also activation of LAP promotes tumour immune tolerance. LAP-sufficient tumour animal models revealed accumulation of M2 macrophages which support the pro-tumorigenic effects of tumour-associated macrophages (TAMs) [33]. Consequently, T cell differentiation is skewed towards regulatory T cells that support inflammation resolution [19, 33]. Indeed, LAP-deficient TAMs trigger STING-mediated type I interferon responses inducing a pro-inflammatory gene expression and increasing CD8^+^ T cell function. Remarkably, the overexpression of Rubicon in cancer patients, which is required for LAP but not autophagy, have been suggested as a potential poor prognostic marker [51]. In line with that, evidence suggest that specifically targeting LAP within the tumour microenvironment through pharmaceutical means promotes an anti-tumour response in a T cell-dependent manner [33]. Hence, development of therapies targeting efferocytosis-related pathways, in macrophages as well as neutrophils, could present a promising approach for cancer treatment.

Neutrophils support tumour growth and spreading not only in the tissue but also in the blood stream. They form heterologous clusters with circulating tumour cells (CTCs) which prolongs their half-life (Fig. 2) [52]. CTC-neutrophils clusters support cell cycle progression, proliferation and survival of tumour cells resulting in extended metastatic potential [53]. Patients with CTC–neutrophil clusters have poorer outcomes compared to those with homotypic CTC clusters [54]. CTC-neutrophil clusters may also include NETs, which promote adhesion and extravasation of CTCs at the site of metastasis [55, 56]. However, sequencing analysis of CTC-associated neutrophils revealed a N2-like gene expression profile, indicating that not all neutrophils in the clusters form NETs [41]. N2 neutrophils offer CTCs protection from immune surveillance by inhibition of CD8^+^ T cells NK cells [57]. The blood-stream is a harsh environment for CTCs and single tumour cells may die quickly. They may be efferocytosed by adjacent neutrophils in the cluster, leading to mitogenic support of remaining tumour cell in the cluster. In summary, anti-inflammatory neutrophils support tumour growth and spreading in a variety of ways. Although there is growing evidence that efferocytosis contributes to tumorigenic TAN polarization, further research is needed to confirm this concept.

## Conclusion and outlook

There is ample evidence that efferocytosis by neutrophils plays an important role in the response to dead cell accumulation. During inflammation, they contribute to the clearance of aggregates of apoptotic neutrophils. In cancer, they participate in the removal of dead tumour cell aggregates. The neutrophil response after engulfing apoptotic cells contributes to the resolution of inflammation and tissue regeneration. However, in the case of cancer, this can be harmful. A meta-analysis of expression signatures from more than 18,000 human tumours found that neutrophils are the tumour-associated cell type linked with the worst prognosis [58]. Neutrophilic efferocytosis might contribute to this situation. As professional phagocytes, neutrophils have the full machinery for engulfment and express many receptors for the detection and binding of dead cells. However, it is also still unclear whether neutrophils distinguish between the different types of cell death from which their target cells have died. Although an increasingly clear picture is emerging on efferocytosis by neutrophils, there are still many unanswered questions awaiting exploration.

## Data Availability

Data sharing not applicable to this article as no datasets were generated or analyzed during the current study.
